# Awareness, Barriers and Concerns of Adolescents Toward the COVID-19 Vaccine: A Cross-Sectional Study in Singapore

**DOI:** 10.3389/fpubh.2022.903152

**Published:** 2022-06-28

**Authors:** Prawira Oka, Benecia Wan Qing Thia, Shyna Zhuoying Gunalan, Janae Rae Yann Kwan, Ding Xuan Ng, Wai Keong Aau, Juan Dee Wee, Ngiap Chuan Tan

**Affiliations:** ^1^SingHealth Polyclinics, Singapore, Singapore; ^2^SingHealth-Duke NUS Family Medicine Academic Clinical Program, Singapore, Singapore; ^3^Centre for Science Research and Talent Development (CENTAD), Hwa Chong Institution, Singapore, Singapore

**Keywords:** adolescents, awareness, barriers, COVID-19, perceptions, vaccination

## Abstract

**Background:**

COVID-19 vaccination is a key public health measure to mitigate the crippling effects of the pandemic. COVID-19 vaccination began in adults and targeted adolescents at a later stage. However, medical decision-making on its uptake among the latter was unknown, which could be affected by their literacy and concerns about the vaccine. The study aimed to elucidate the awareness, concerns and barriers of adolescents toward the COVID-19 vaccine.

**Methods:**

A cross-sectional online survey using a self-developed questionnaire was conducted between June to November 2021. The adolescent participants were students from institutes of post-secondary education who were recruited via convenience sampling. The data were collated from an officially approved electronic platform, audited and analyzed. Continuous and categorical variables were expressed as mean (standard deviation) and percentages, respectively.

**Results:**

A total of 460 adolescents participated in the study (mean age: 16.99 ± 0.99; 73% female). Most of them (91.5%) were aware of the COVID-19 vaccines. The main barriers to vaccination were uncertainty about long-term health risks (38.6%) and vaccine efficacy (37.3%). Regarding the potential vaccine side effects, they were concerned about: anaphylactic shock (73.2%), low blood pressure (58.3%) and fast heartbeat (58.0%). Only 58% expressed willingness for repeated COVID-19 vaccination.

**Conclusion:**

Despite high COVID-19 vaccine awareness, the adolescents were concerned about the potentially severe but rare side-effects. The study identified apprehension regarding vaccine efficacy and the potential long-term health impact as the main barriers to vaccination. Future studies should address these concerns to scale vaccination programs among adolescents.

## Introduction

Since the outbreak of the COVID-19 pandemic and the emergence of various strains, vaccinations have emerged as a key solution to curb its spread. COVID-19 vaccinations have been proven to reduce both the incidence and severity of infections (1, 2). The CDC published data that fully vaccinated individuals were 5 times less likely to become infected and more than 10 times less likely to require hospitalization or succumb to COVID-19 (3). Some of the barriers to vaccination include accessibility and vaccine hesitancy.

Vaccine hesitancy is defined as the delay in acceptance or vaccination refusal despite its availability (4). Rates of COVID-19 vaccine hesitancy of up to 35% and 58% have been reported in the US and China (5, 6). Factors commonly associated with vaccine hesitancy include concerns over efficacy, adverse effects, safety and cost (7, 8). It is crucial to elucidate the awareness, concerns and barriers to addressing vaccine hesitancy. Presently information is lacking on how the youth in Singapore perceive the COVID-19 vaccine.

Vaccinations were initially approved for individuals aged 18 years and above. On 10^th^ May 2021, the Pfizer^®^ COVID-19 vaccination received Food and Drug Agency (FDA) approval for use in individuals aged 12 years and older (9). With individuals under 20 years of age constituting nearly 20% of Singapore's population, it is critical that those eligible are vaccinated promptly to scale up the herd immunity (10).

At the beginning of this study on 1^st^ June 2021, the vaccination rate among adults was 34% (11). In contrast, 2% of individuals between 12 and 19 years of age had completed the full regime due to phased-in vaccination for this age group. Almost all of these youths are students in schools, with those aged 16 years and older studying in institutes of higher learning such as junior colleges (or the equivalent of high schools), polytechnics and institutes of technical education. Cai et al. (12) found that 48.3% of polled Chinese adolescents were aware that COVID-19 vaccines could provide protection. Similar to adults, a study conducted among adolescents in Hong Kong reported concerns over vaccine safety (79%) and efficacy (52%) as barriers to COVID-19 vaccination (13). Information on the awareness, concerns and barriers preventing adolescents in Singapore from receiving the COVID-19 vaccine remains scarce despite its well-educated population.

Ruggeri et al. (14) reported that children and adolescents made different choices in medical decision-making. According to the authors, adolescents want to be responsible for their own medical decision, instead of their parents. They also highlighted the need to explore better approaches to involve adolescents in their medical decision-making process. The uptake of the COVID-19 vaccine requires medical decision-making but the process of reaching such a decision among older adolescents has yet to be determined. Understanding the perspectives of adolescents in this age group will allow public health policymakers to assess their eventual COVID-19 vaccine uptake.

Hence, this survey aimed to determine the willingness of older adolescents to take up the COVID-19 vaccine and to identify their concerns, awareness and barriers to the vaccine in Singapore.

## Materials and Methods

### Study Design

This cross-sectional study was implemented as a web-based questionnaire survey. The online survey eased the dissemination to the target participants without physical contact in a pandemic, which also allowed participants to complete the survey anonymously without any undue external pressures.

### Population

This study was conducted among adolescents aged between 16 and 17 years of age to determine their concerns, awareness and deterring factors toward the COVID-19 vaccine. They were post-secondary students in junior colleges, polytechnics and Institutes of Technical Education (ITE). Junior colleges are the equivalent of high schools, whereas polytechnics provide more practice-based learning experiences culminating in a diploma. ITE provides technical and vocational education (15).

### Questionnaire Design

The survey consisted of 6 domains: demographic, history of severe allergic reaction to vaccines or medications, knowledge and attitude on the vaccine, concerns about the vaccination, factors influencing their vaccine uptake and willingness to proceed with COVID-19 vaccinations. Participants filled in their gender, ethnicity, year of birth and sites of Institute of Higher Learning but no participant identifier was collected. The questionnaire allowed them to respond by selecting either binary (Yes/No) or multiple allowable options. The questionnaire underwent face validation by a group of polytechnic students in an unpublished study.

### Awareness of the COVID-19 Vaccine

Awareness of common facts about the COVID-19 vaccine was assessed via questions regarding its efficacy, potential side-effects and cost.

### Concerns About the COVID-19 Vaccine

Concerns about the COVID-19 vaccine side-effects were determined by asking participants about their concerns regarding specific side-effects. The side-effects ranged from common but mild symptoms such as tiredness to more severe side-effects such as anaphylactic shock.

### Barriers to COVID-19 Vaccination

Barriers to COVID-19 vaccination were assessed via questions encompassing vaccine accessibility, vaccine administration and COVID-19 vaccine-specific factors including efficacy and potential long-term side-effects.

### E-Survey Design and Subject Recruitment

The e-survey was conducted from 1^st^ June to 30^th^ November 2021. The study team formulated a preliminary set of survey questions using Google Forms. After the finalization of the survey questions on Google Forms, the final survey was then created using the form builder tool called “FormSG,” which is a national survey platform to create digital government forms developed by Singapore's GovTech's Data Science & Artificial Intelligence Capability Centre. After the form is built-in FormSG, it is identified *via* a unique URL weblink. With the URL weblink, the corresponding QR code is created as well. The QR code will facilitate signing in to complete the survey simply just by scanning it via the application on mobile phones. Otherwise, the participants were able to access the survey via the weblink. The link and QR code were generated from FormSG and distributed to the students through snowball convenience sampling via Whatsapp, Telegram, emails and word of mouth. The submissions of the individual responses were collated via a dedicated email as pre-set in the FormSG. At the end of the study, all the individual email files were exported from Outlook as.pst file format. The.pst file format was next imported into the Data Collation tool in FormSG where it was converted into Excel. The data was next exported for analysis and visualization.

### Statistical Analysis

Descriptive statistics were done to describe the sample and associations performed to consider relationships between gender and ethnicity with concerns and willingness to get COVID-19 booster jabs. Continuous and categorical variables were expressed as mean (standard deviation) and percentages, respectively.

## Results

### Subject Demographics

The demographic characteristics are shown in Table 1. A total of 460 adolescents aged between 16 and 17 participated in this study, most were female (73.0%) and Chinese (90.0%) with an average age of 16.99 years.

**Table 1:** Subject demographics (*n* = 460).

**Demographics**	**Mean (SD) or *n* (%)**
Age, mean (SD)	16.99 (0.09)
**Institution**
Institute of technical education	8 (1.7)
Polytechnic	28 (6.1)
Junior college	424 (92.2)
**Gender**, ***n*** **(%)**
Male	124 (27.0)
Female	336 (73.0)
**Ethnicity**, ***n*** **(%)**
Chinese	414 (90.0)
Non-Chinese	46 (10.0)

### Awareness of COVID-19 Vaccine

Table 2 depicts the awareness of the COVID-19 vaccine among adolescents. Most were aware that vaccines were not 100% effective (98%), could potentially result in minor side-effects (98%), were free for citizens and permanent residents (96%), effective after two doses (95%) and did not cause genetic modification (92%).

**Table 2:** COVID-19 vaccination awareness (*n* = 460).

**Vaccination awareness**	***n* (%)**
**Vaccine not 100% effective**
Yes	452 (98.3)
No	8 (1.7)
**Minor side-effects**
Yes	449 (97.6)
No	11 (2.4)
**Free (Singaporeans)**
Yes	443 (96.3)
No	17 (3.7)
**Effective after two doses**
Yes	435 (94.6)
No	25 (5.4)
**No modifications to genes**

### Awareness and Concerns of Vaccine Side-Effects

Table 3 depicts the awareness and concerns of the specific vaccine side-effects among adolescents. They were least aware of uncommon side-effects such as fast heartbeat (19.6%), anaphylactic shock (18.9%) and low blood pressure (14.3%). The majority of them were most concerned about anaphylactic shock (73.2%), fast heartbeat (58.0%) and low blood pressure (58.3%).

**Table 3:** Awareness and concerns regarding side-effects of the COVID-19 vaccine.

	***n*** **(%)**	
**Symptoms**	**Vaccine symptom**	**Vaccine symptom**
	**awareness**	**concern**
**Anaphylactic shock**
Yes	87 (18.9)	337 (73.2)
No	373 (81.1)	123 (26.8)
**Fast heartbeat**
Yes	90 (19.6)	267 (58.0)
No	370 (80.4)	193 (42.0)
**Low blood pressure**
Yes	66 (14.3)	268 (58.3)
No	394 (85.7)	192 (41.7)
**Fever**
Yes	410 (89.1)	203 (44.1)
No	50 (10.9)	257 (55.9)
**Nausea**
Yes	257 (55.9)	145 (31.5)
No	203 (44.1)	315 (68.5)
**Headache**
Yes	362 (78.7)	131 (28.5)
No	98 (21.3)	329 (71.5)
**Muscle pain**
Yes	338 (73.5)	97 (21.1)
No	122 (26.5)	363 (78.9)
**Redness/Swelling**
Yes	257 (55.9)	79 (17.2)
No	203 (44.1)	381 (82.8)
**Chills**
Yes	207 (45.0)	80 (17.4)
No	253 (55.0)	380 (82.6)
**Tiredness**
Yes	368 (80.0)	75 (16.3)
No	92 (20.0)	385 (83.7)

### Barriers to COVID-19 Vaccination

**Figure 1:**
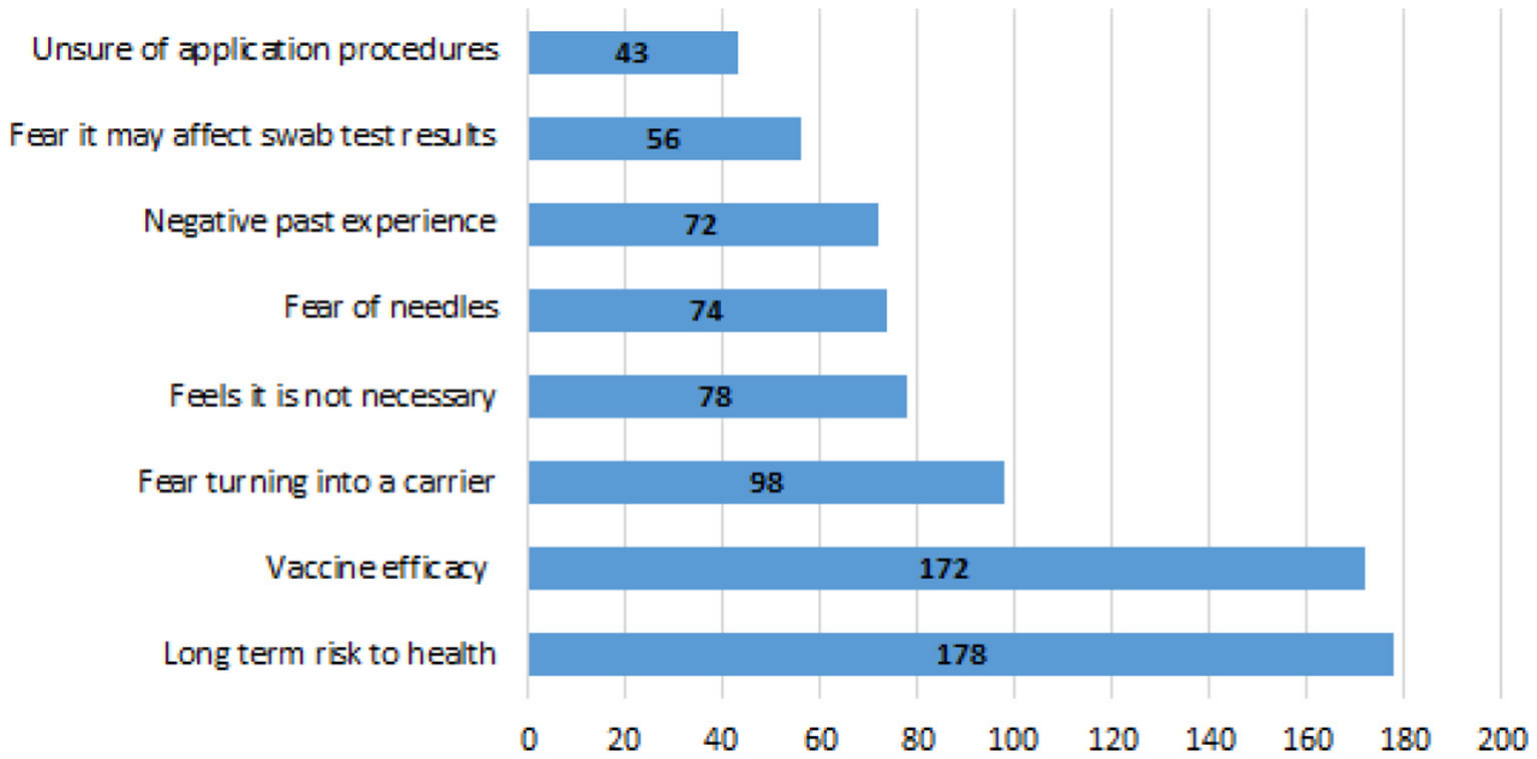
Deterring factors to vaccination.

Figure 1 shows the deterring reasons to vaccination. Vaccine efficacy (37.4%) and risk to long-term health (38.7%) were the most common deterring reasons to vaccination. A small proportion of adolescents (9.3%) responded that they were not sure about the application procedures as a reason for not getting vaccinated.

### Willingness to Undergo Repeated Vaccinations

Overall, 265 (58%) adolescents were willing to repeat the vaccination, 33 (7%) were reluctant and the remaining 162 (35%) were ambivalent.

### Sources of Information

**Figure 2:**
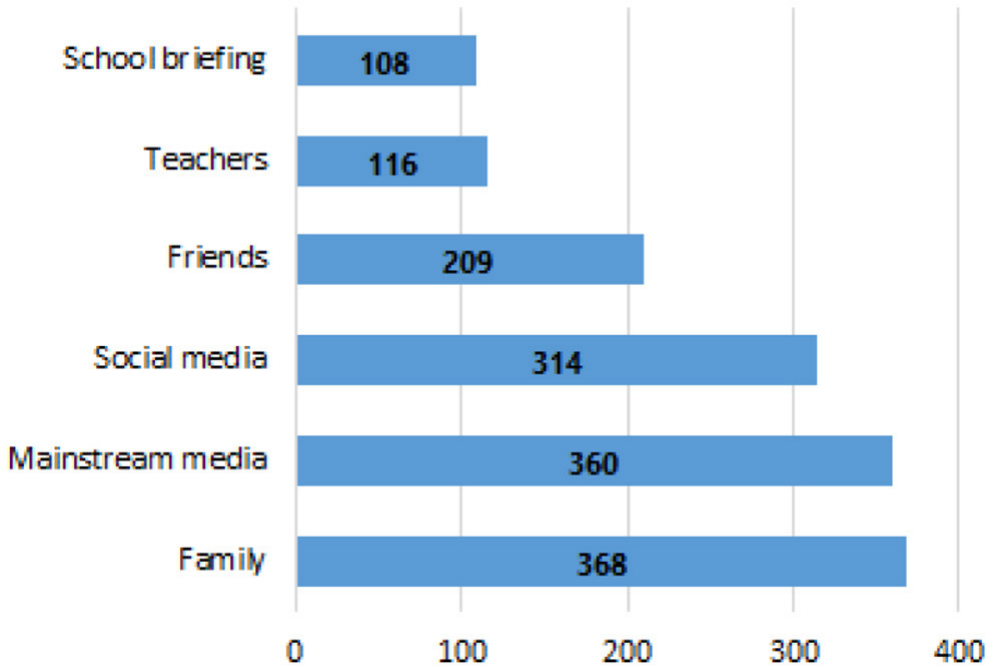
Source of vaccination information.

Figure 2 displays the source of vaccination information, most participants obtained vaccination information from their families (80.0%). The least reported sources of information were teachers (25.2%) and school briefings (23.5%).

## Discussion

The surveyed adolescents displayed high literacy of COVID-19 vaccines, such as vaccine efficacy, its side-effects (including non-interference with DNA), cost and implementation for local residents. In contrast, a 2020 survey by the Nanyang Technical University of Singapore showed one in four Singaporeans falsely believed that the COVID-19 vaccine alters DNA (16). Unfortunately, their awareness did not include other uncommon COVID-19 vaccine-related adverse effects. About 19% of respondents were aware of the potential for the vaccine to cause a fast heartbeat and anaphylactic shock. Unsurprisingly, the potential adverse effects with the least awareness were those that these adolescents were most concerned with, reflecting the need for further deliberations. While these concerns should be addressed, it is important to strike a good balance between ensuring that individuals obtain the necessary information to make an informed decision without inducing unnecessary fear that may adversely affect vaccination rates.

Vaccine hesitancy was related to its perceived efficacy (37.4%) and effect on long-term health (38.7%). The vaccine has been proven to be efficacious with current available data not suggesting any known long-term detrimental effects to one's health (1). More adolescents were concerned about vaccine efficacy than the 24% reported by a US study conducted among college students (17). Interestingly, the concerns over vaccine efficacy are in contrast to the good awareness that many respondents had regarding the efficacy of vaccination. This reveals a gap between the knowledge and its application. These concerns need to be addressed and identifying the best avenue to do so is imperative.

Most adolescents reported obtaining information from family (80.0%) and mainstream media (78.2%). In contrast, around 25% reported obtaining information from teachers and school briefings. With adolescents spending extended periods of their day in school, the education professionals should not miss the opportunity to provide them with the requisite knowledge and opportunity to clarify any doubts regarding the COVID-19 vaccine.

The study revealed that only 58% of participants were willing to undergo repeated COVID-19 vaccinations to maintain their immunity. As of 10^th^ February 2022, 96% of adolescents aged 12 to 19 years of age received their primary doses of COVID-19 vaccines (11). The difference between the survey results and the actual vaccination rates could be explained by vaccine approval and government-led campaigns. On 10^th^ June 2021, the Pfizer ^®^ COVID-19 vaccine became the first vaccine approved for use in Singapore for individuals aged 12 to 17 years of age (18). This presented the only available vaccination option for youths in this age group. Should they be medically ineligible to receive the Pfize^®^ vaccine, they would be eligible to receive the Sinovac^®^ vaccine via the Pediatric Sinovac^®^ after mRNA (SAM) public health program (19). Access to the Sinovac^®^ vaccine is regulated because the Singapore Ministry of Health Expert Committee determined that it was lower in efficacy compared to the Pfizer^®^ vaccine in addition to the lack of World Health Organization (WHO) approval for use in this population (19, 20). Locally, the inability to opt for a non-mRNA vaccine option in this age group does not seem to have significantly affected the vaccine uptake rates. Apart from vaccine availability, government-led campaigns such as VacciNationSg were introduced in March 2021 to improve public awareness of the COVID-19 vaccination (21). Following the approval of the Pfizer^®^ vaccine for use in those aged 12 to 17 years of age, specific material aimed at this demographic was disseminated via not only traditional but also digital platforms including TikTok, Twitter and Instagram. The impact of these measures may account for the vaccination rate being higher than the 57% reported by the United States in youths aged 12 to 17 years of age (22).

Following the positive uptake of primary COVID-19 vaccinations, booster vaccinations will be required as immunity wanes with time. National healthcare policies were introduced to foster positive uptake of the booster through the Booster Vaccination Program. The government announced on 21^st^ January 2022 that individuals aged 12 years and above would only be considered fully vaccinated once they receive the booster vaccination with effect from 14^th^ March 2022 (23). Once eligible, parents of individuals between 12 and 17 years of age received a text message to schedule their booster vaccination. Since November 2021, vaccine-differentiated Safe Management Measures (SMMs) aimed to reduce the risk to non-fully vaccinated individuals by restricting their access to high-risk settings. These measures include being unable to enter shopping centers or dine-in restaurants (24). Individuals may be motivated to receive the booster dose to avoid inconveniencing their daily activities. These policies incentivize the public to comply with vaccination recommendations for the collective good of the populace.

### Strengths and Limitations

The use of an electronic questionnaire survey is a strength of this study. An e-survey offers a cost-free and convenient recruitment method to reach out to adolescents, who are likely to be tech-savvy and comfortable with navigating the internet in a highly digitalized community like Singapore. Using a secured and officially approved digital platform that local residents are familiar with, enhances the confidence of the adolescents to participate in the survey.

Nonetheless, the data was collected via an e-survey, which could have resulted in more responses from motivated individuals who participated in the study. The sampling can be skewed as reflected by the higher proportion of female and Chinese ethnicity compared to the baseline demographics expected in this age group.

### Future Work

The COVID-19 vaccination uptake thus far has been promising with 96% of youth between 12 and 19 years of age having completed the primary vaccination (11). With the next phase being booster vaccinations, armed with the information gathered in this survey, the VacciNationSG campaign can be further tailored to address some of the concerns and barriers to vaccination with the delivery of information utilizing platforms such as schools. Supplementary services such as hotlines and online portals can be further promoted to provide individuals with accurate information promptly.

## Conclusion

The study identified concerns and barriers to vaccination with only slightly more than half of the participants being willing to undergo repeated vaccinations. Overall the local adolescents had high COVID-19 vaccine awareness with concerns over the potentially severe, albeit rarer, side-effects. Apprehension regarding vaccine efficacy and the potential long-term impact on health were the main barriers to vaccination and needs to be addressed promptly to facilitate possible repeat vaccination when robust evidence becomes available in the future.

## Data Availability Statement

The raw data supporting the conclusions of this article will be made available by the authors, without undue reservation.

## Ethics Statement

The studies involving human participants were reviewed and approved by Institutional Review Board at Hwa Chong Institution. Written informed consent from the participants' legal guardian/next of kin was not required to participate in this study in accordance with the national legislation and the institutional requirements.

## Author Contributions

BT, SG, JK, JW, and NT conceptualized the study. BT, SG, JK, and JW implemented the e-survey. WA extracted the survey data. DN and WA performed the data analysis. PO, BT, SG, JK, DN, WA, and NT interpreted the results. PO and NT drafted the manuscript. All authors reviewed and approved the final manuscript.

## Funding

This study was supported in-kind (research expertise) by the Research Department in SingHealth Polyclinics. The publication cost was supported by the SingHealth Polyclinics – Centre Grant.

## Conflict of Interest

The authors declare that the research was conducted in the absence of any commercial or financial relationships that could be construed as a potential conflict of interest.

## Publisher's Note

All claims expressed in this article are solely those of the authors and do not necessarily represent those of their affiliated organizations, or those of the publisher, the editors and the reviewers. Any product that may be evaluated in this article, or claim that may be made by its manufacturer, is not guaranteed or endorsed by the publisher.

## References

[1] Baden LR El Sahly HM Essink B Kotloff K. Efficacy and safety of the mRNA-1273 SARS-CoV-2 vaccine. N Engl J Med. (2021) 384:403–16. 10.1056/NEJMoa2035389 33378609 PMC7787219

[2] Food Drug Administration. Pfizer-Biontech COVID-19 Vaccine (BNT162, PF-07302048) Vaccines and Related Biological Products Advisory Committee Briefing Document. From Food and Drug Administration. (2020). Available online at: https://www.fda.gov/media/144246/download (accessed January 16, 2022).

[3] Scobie H Johnson A Suthar A Severson R Alden N Balter S. Monitoring incidence of COVID-19 cases, hospitalizations, and deaths, by vaccination status — 13 U.S. jurisdictions, April 4–July 17, 2021. MMWR Morb Mortal Wkly Rep. (2021) 70:1284–90. 10.15585/mmwr.mm7037e1 34529637 PMC8445374

[4] MacDonald NE. Vaccine hesitancy: definition, scope and determinants. Vaccine. (2015) 33:4161–4. 10.1016/j.vaccine.2015.04.036 25896383

[5] Daly M Jones A Robinson E. Public trust and willingness to vaccinate against COVID-19 in the US from october 14, 2020, to march 29, 2021. JAMA. (2021) 325:2397–9. 10.1001/jama.2021.8246 34028495 PMC8145162

[6] Gao X Li H He W Zeng W. COVID-19 vaccine hesitancy among medical students: the next COVID-19 challenge in Wuhan, China. Disaster Med Public Health Prep. (2021) 1–6. 10.1017/dmp.2021.291 34496990 PMC8564029

[7] Al-Mulla R Abu-Madi M Talafha Q Tayyem R Abdallah A. COVID-19 vaccine hesitancy in a representative education sector population in Qatar. Vaccines. (2021) 9:665. 10.3390/vaccines9060665 34207012 PMC8235273

[8] Lin Y Hu Z Zhao Q Alias H Danaee M Wong L. Understanding COVID-19 vaccine demand and hesitancy: a nationwide online survey in China. PLOS Negl Trop Dis. (2020) 14:e0008961. 10.1371/journal.pntd.0008961 33332359 PMC7775119

[9] Food and Drug Administration. Pfizer- BioNTech COVID-19 Vaccine EUA Letter of Authorization. from Food and Drug Administration: Pfizer- BioNTech COVID-19 Vaccine EUA Letter of Authorization. (2021) (accessed January 6, 2022).

[10] Department of Statistics Singapore. Elderly, Youth and Gender Profile. (2021). Available online at: http://singstat.gov.sg/find-data/search-by-theme/population/elderly-youth-and-gender-profile/latest-data (accessed January 6, 2022).

[11] Ministry of Health Singapore. Ministry of Health Singapore. Available online at: https://www.moh.gov.sg/covid-19/vaccination/statistics (accessed January 10, 2022).

[12] Cai H Bai W Liu S Liu H Chen X Qi H. Attitudes toward COVID-19 vaccines in Chinese adolescents. Front Med. (2021) 8:691079. 10.3389/fmed.2021.691079 34307416 PMC8292666

[13] Wong W Leung D Chua G Duque J Peare S So H. Adolescents' attitudes to the COVID-19 vaccination. Vaccine. (2022) 967–9. 10.1016/j.vaccine.2022.01.010 35063284 PMC8752287

[14] Ruggeri A Gummerum M Hanoch Y. Braving difficult choices alone: children's and adolescents' medical decision making. PLoS ONE. (2014) 9:e103287. 10.1371/journal.pone.0103287 25084274 PMC4118856

[15] Ministry of Education Singapore. Overview of Post-Secondary Education Institutions (PSEIs). Ministry of Education Singapore. Available online at: https://www.moe.gov.sg/post-secondary/overview (accessed November 26, 2021).

[16] Qing A. Around 1 in 4 Singapore residents surveyed believe false claim that Covid-19 vaccine alters DNA. The Straits Times (2020). Available online at: https://www.straitstimes.com/singapore/around-one-in-four-singapore-residents-surveyed-believe-false-claim-about-covid-19-vaccine (accessed January 20, 2022).

[17] Silva J Bratberg J Lemay V. COVID-19 and influenza vaccine hesitancy among college students. J Am Pharm Assoc. (2021) 61:709–14. 10.1016/j.japh.2021.05.009 34092517 PMC8139529

[18] Ministry of Health Singapore. Updates on COVID-19 National Vaccination Programme. (2021). Available online at: https://www.moh.gov.sg/news-highlights/details/updates-on-covid-19-national-vaccination-programme_24Jun2021 (accessed January 8, 2022).

[19] Ministry of Health Singapore. Non-mRNA Vaccines. (2022). Available online at: https://www.moh.gov.sg/news-highlights/details/non-mrna-vaccines (accessed February 25, 2022).

[20] World Health Organization. The Sinovac-CoronaVac COVID-19 vaccine: What you Need to Know. (2021). Available online at: https://www.who.int/news-room/feature-stories/detail/the-sinovac-covid-19-vaccine-what-you-need-to-know (accessed February 25, 2022).

[21] Lai L. VacciNationSG Campaign Launched to Raise Awareness of Covid-19 Vaccine, Combat Misinformation. The Straits Times (2021). Available online at: https://www.straitstimes.com/singapore/vaccinationsg-campaign-launched-to-raise-awareness-of-covid-19-vaccine-combat (accessed February 8, 2022).

[22] The New York Times. See How Vaccinations Are Going in Your County and State. (2020). Available online at: https://www.nytimes.com/interactive/2020/us/covid-19-vaccine-doses.html#age (accessed February 25, 2022).

[23] Tan C. Those Aged 12 to 17 Must Take Covid-19 Booster Dose From March 14 to Be Deemed Fully Vaccinated. The Straits Times (2022). Available online at: https://www.straitstimes.com/singapore/youths-aged-12-to-17-must-take-booster-to-be-fully-vaccinated-from-march-14 (accessed January 22, 2022).

[24] Ministry of Health Singapore. COVID-19 PHASE ADVISORY. Available online at: https://www.moh.gov.sg/covid-19-phase-advisory (accessed March 7, 2022).

